# Characterization of Trapped Lignin-Degrading Microbes in Tropical Forest Soil

**DOI:** 10.1371/journal.pone.0019306

**Published:** 2011-04-29

**Authors:** Kristen M. DeAngelis, Martin Allgaier, Yaucin Chavarria, Julian L. Fortney, Phillip Hugenholtz, Blake Simmons, Kerry Sublette, Whendee L. Silver, Terry C. Hazen

**Affiliations:** 1 Earth Sciences Division, Ecology Department, Lawrence Berkeley National Lab, Berkeley, California, United States of America; 2 Microbial Communities Group, Deconstruction Division, Joint BioEnergy Institute, Emeryville, California, United States of America; 3 Microbial Ecology Program, DOE Joint Genome Institute, Walnut Creek, California, United States of America; 4 Sandia National Lab, Livermore, California, United States of America; 5 Center for Applied Biogeosciences, University of Tulsa, Tulsa, Oklahoma, United States of America; 6 Department of Environmental Science, Policy and Management, University of California, Berkeley California, United States of America; University of Illinois at Urbana-Champaign, United States of America

## Abstract

Lignin is often the most difficult portion of plant biomass to degrade, with fungi generally thought to dominate during late stage decomposition. Lignin in feedstock plant material represents a barrier to more efficient plant biomass conversion and can also hinder enzymatic access to cellulose, which is critical for biofuels production. Tropical rain forest soils in Puerto Rico are characterized by frequent anoxic conditions and fluctuating redox, suggesting the presence of lignin-degrading organisms and mechanisms that are different from known fungal decomposers and oxygen-dependent enzyme activities. We explored microbial lignin-degraders by burying bio-traps containing lignin-amended and unamended biosep beads in the soil for 1, 4, 13 and 30 weeks. At each time point, phenol oxidase and peroxidase enzyme activity was found to be elevated in the lignin-amended versus the unamended beads, while cellulolytic enzyme activities were significantly depressed in lignin-amended beads. Quantitative PCR of bacterial communities showed more bacterial colonization in the lignin-amended compared to the unamended beads after one and four weeks, suggesting that the lignin supported increased bacterial abundance. The microbial community was analyzed by small subunit 16S ribosomal RNA genes using microarray (PhyloChip) and by high-throughput amplicon pyrosequencing based on universal primers targeting bacterial, archaeal, and eukaryotic communities. Community trends were significantly affected by time and the presence of lignin on the beads. Lignin-amended beads have higher relative abundances of representatives from the phyla Actinobacteria, Firmicutes, Acidobacteria and Proteobacteria compared to unamended beads. This study suggests that in low and fluctuating redox soils, bacteria could play a role in anaerobic lignin decomposition.

## Introduction

There is a strong impetus both nationally and internationally for devising new, non-fossil based fuels that are generated in a sustainable way with minimum greenhouse gas production [1]. Plant biomass derived from either crop waste or dedicated feedstocks such as switchgrass (*Panicum virgatum*) could potentially provide energy via biofuels if a system for unlocking this energy were devised that was robust, efficient and inexpensive [2]. One hurdle in cellulosic biofuels engineering is the presence of lignin, which can comprise up to 25% of plant biomass in herbaceous plants [3]. While pretreatment eliminates most of the lignin during biofuels production, lignin can pose a challenge due to its ability to inhibit cellulosic enzymes and as a potentially viable waste feedstock [4], [5].

Lignin is a complex heteropolymer linked to cellulose, giving plants structural integrity. The deconstruction of lignin and its dissociation from cellulose presents a challenge for soil microbes and biofuels engineers alike. The repeating units of phenolic monomers, *p*-coumaryl alcohol, coniferyl alcohol, and sinapyl alcohol, are synthesized in different ratios and combinations depending upon the type of plant, and so conferring its structural characteristics. The best understood mechanism for breaking open the rings in the lignin phenols belongs to fungi, specifically via oxygen free radical attached by the enzymes dioxygenases [6], generally requiring oxic conditions. The known potential lignin-degrading bacteria are mostly derived from guts of wood-eating insects and include Alphaproteobacteria, Gammaproteobacteria and Actinomycetes [7], with the best-characterized being *Streptomyces viridosporus* [8]. Phenol-degrading bacteria such as *Kocuria* and *Staphylococcus* [9], peroxidase-producing *Flavobacterium meningosepticum* [10], and bacterial degraders of polyaromatic hydrocarbons [6] may also have a natural ability for degrading lignin derived from decomposing plant biomass. Discovery of novel anaerobic bacterial lignin-degrading enzymes would be beneficial to the industrial production of next-generation biofules, due to their potential application to microbial engineered biofuels-producing organisms, lack of requirement of oxygen, and range of specificity or environmental conditions.

Plant litter quality is a key controller of decomposition rates in soils, and lignin and the lignin∶N ratio play a particularly important role in late stage decomposition [11], [12]. Humid tropical forest soils have the fastest rates of above- and belowground plant litter decomposition globally [12]. Near complete decomposition of a wide range of plant tissues has been recorded over 1–2 years in these ecosystems [12], [13], [14], [15]. This rapid and complete decomposition belowground is surprising given the low and variable redox conditions typical of humid tropical forest soils [16]. The combination of fast decomposition and low and fluctuating redox suggests the presence of efficient anaerobic or facultative lignin-degrading microorganisms in the soils. While generally it is believed that fungi dominate plant decomposition and lignin degradation [17], few fungi are able to tolerate anoxic conditions [18], [19]. Thus, humid tropical forest soils are ideal sites to explore the potential for bacterial lignin degraders.

Humid tropical forest soils house an immense and unexplored microbial diversity [20], extremely high biomass [21], and a microbial community that is very productive and uniquely fueled by the high iron present in these strongly weathered soils [22], [23]. They present an attractive target for discovery of novel enzymes and pathways for deconstruction of plant material and improvement of efficiency of biofuels production derived from cellulosic feedstocks. In this study we used lignin-baited ‘bio-traps’ to investigate the microbes and enzymes responsible for lignin decomposition in Puerto Rico tropical forest soils.

## Methods

### Experimental design

We employed Bio-trap® samplers (Microbial Insights, Inc., Rockford, TN), where pure low-sulfonate alkali lignin (Sigma-Aldrich, #471003) was trapped within Bio-Sep® (University of Tulsa) beads to create the lignin-amended traps, and unamended beads were used as controls. The traps contain about 200 g Bio-Sep® beads, 3 mm diameter balls composed of 75% powdered activated carbon and 25% DuPont aramid polymer, resulting in a matrix that has 75% porosity [24]; the beads are meant to provide a non-reactive surface that is in close contact with the environment, but separate enough to isolate and study. The traps were constructed as beads enclosed in a PVC chamber with holes cut into the sides for exposure to the soil (Figure S1). The bio-traps were buried in the Bisley Research Watershed located in Luquillo Experimental Forest, part of the NSF-sponsored Long Term Ecological Research Project in northern Puerto Rico; the fieldwork was conducted and samples collected and transported under USDA permit number P526P-08-00634. The field site is located in the Tabonuco forest at 350 masl (18°18′N, 65°50′W), and receives approximately 3500 mm of rainfall per year relatively evenly distributed throughout the year, with an average annual temperature of 23°C with little seasonal variation. Soils are deep, highly weathered, clayey Ultisols, rich in Fe and Al oxides and hydroxides [25]. The traps were buried in pairs in the 0–15 cm soil depth. Six biological replicates of each treatment were buried at four locations in the forest, with enough bead traps for destructive sampling at four time points, chosen to capture both initial and late-stage colonization: T1, 1 week; T2, 4 weeks; T3, 13 weeks; T4, 30 weeks (total n = 192). The biological replicates were buried about 2 m from a centroid point, and located 2–4 m from each other. Oxygen sensors were also placed in the 0–15 cm depth (Apogee Instruments) using soil equilibration chambers [26]; oxygen was continuously measured for the duration of the experiment. At each sampling time point, trace gasses were sampled from the headspace of the equilibration chambers as a further indication of the redox environment. Beads were excavated from the field, shipped to the lab overnight at ambient temperatures, and immediately analyzed for enzyme activity or archived at −80°C for microbial community analysis.

### Enzyme assays

Enzyme assays were performed on beads fresh from the field at the 1, 4, and 30 weeks. For enzyme assays, 3–4 g beads were added to 50 mM acetate buffer solution pH 5.5, mixed by stirring for 2 min, then the buffer extract was analyzed for enzyme activity. We performed oxidative enzyme assays using a colorimentric method for phenol oxidase (EC 1.10.3.2) L-dihydroxyphenylalanine (L-DOPA), and peroxidase (EC 1.11.1.7) DOPA plus 0.3% H_2_O_2_. We also performed cellulase enzyme assays using the fluorogenic detection molecule methylumbylliferyl (MUB): MUB-beta-d-glucopyranoside for beta-glucosidase (EC 3.2.1.21); MUB-cellobioside for cellobiohydrolase (EC 3.2.1.91); MUB-beta-xylopyranoside for beta-xylosidase (EC 3.2.1.37); MUB-N-acetyl-beta-glucosaminidase for chitinase (EC 3.2.1.30). Rates are the mean of sixteen technical replicates of amount of substrate evolved per unit time per gram bead.

### PhyloChip bacterial community analysis and QPCR

DNA from the beads was extracted using a modified CTAB extraction method as previously described [22]. Briefly, the beads were added to CTAB extraction buffer and phenol in Lysing Matrix E tubes (Qbiogene), bead beaten in a FastPrep instrument (Bio101), followed by a chloroform extraction, isopropanol precipitate, and the AllPrep DNA/RNA extraction kit (Qiagen). Quantitative PCR was performed to analyze the total number of bacteria present in each sample. The primer pair used for QPCR was 338F [27] and 518R [28] at an annealing temperature of 53°C. The reaction conditions were otherwise the same as reported for generation of PCR products as for PhyloChip analysis. Bacterial 16S ribosomal RNA genes were PCR amplified in a BioRad iCycler (BioRad Laboratories, Hercules CA) with 10 ng of template per reaction, determined by electrophoresis and verified spectrophotometrically (Nanodrop Technologies, Wilmington, DE). Small subunit (SSU) rRNA gene sequences were amplified using the primer pair 8F/1492R [29], [30] as previously described [22]. For application onto the high-density 16S rDNA microarray (PhyloChip), PCR products were concentrated to 500 ng in 40 µl, then fragmented, biotin labeled and hybridized as previously described [31]. The microbial community analysis was resolved as a subset of 8743 taxa with corresponding hybridization scores reported as arbitrary units (au). Each taxon consists of a set of 25–30 perfect match-mismatch probe pairs. For a taxa to be reported in this analysis, 90% of probe pairs in its set (positive fraction (pf)>0.9) must: (1) have perfect match intensity at least 1.3 times the mismatch, and (2) have both perfect match and mismatch 500-fold above background. Hybridization scores are an average of the difference between perfect match and mismatch fluorescent intensity of all probe pairs excluding the highest and lowest. Final hybridization scores were normalized to total intensity for each PhyloChip.

### Amplicon pyrosequencing

The same four biological replicates were sequenced for small subunit (SSU) rRNA genes using high-throughput amplicon pyrosequencing. The universal primers 926F (5′-aaactYaaaKgaattgacgg-3′) and 1392R (5′-acgggcggtgtgtRc-3′) were used to amplify the V8 variable region of the 16S rRNA gene from bacteria and archaea as well as the 18S rRNA gene in eukarya [32]. The sequences shown do not include adaptor or barcode sequences, and the reverse primer included a 5 bp barcode for multiplexing of samples during sequencing. Emulsion PCR and sequencing of the PCR amplicons was performed following manufacturer's instructions for the Roche 454 GS FLX Titanium technology, with the exception that the final dilution was 1e-8. Sequencing tags were analyzed using the software tool PyroTagger (http://pyrotagger.jgi-psf.org/), which filters by removing low-quality sequences from the set based on the qual file, trims using a 225 bp sequence length threshold, dereplicates, clusters at the OTU level based on 97% identity, then classifies [33]. Classification was based on the greengenes database of ribosomal RNA genes [34] for bacterial and archaeal amplicons, and the SILVA database for eukaryotic amplicons [35]. Because of incomplete classification in some sequences in these databases, not all sequences are classified to the species level.

### Data analysis

The experimental design included six biological replicates, though due to the cost and difficulty of some analyses, four replicates were chosen at random; these cases are stated. Lignin-amended beads and unamended beads were treated as paired samples, since they were buried side-by-side in the soil. To test differences in enzyme activity and relative abundance of taxa by PhyloChip and amplicon pyrosequencing results in the lignin-amended versus unamended beads, student's paired t-tests were performed and evaluated to a p value of 0.05, unless otherwise noted. Richness was based on a presence-absence cutoff using a probe fraction of 0.9, meaning that 90% of the probes that define a taxon must have passed detection [36]. Ordination of whole community detected by PhyloChip or pyrosequencing was performed using non-metric multidimensional scaling with Bray-Curtis distance measure [37]. The software package Phylocom v 4.0.1 was used to analyze the phylogenetic dispersion of the PhyloChip microbial communities [38]. There are two measures: net relatedness index (NRI) and nearest taxon index (NTI), and in both indexes, positive values indicate clustering compared to the null model, in which species in each sample become random draws from the phylogenetic pool. The NRI is based on mean phylogenetic distance across the whole community, and is more positive when the overall microbial community is more phylogenetically clustered than the null model, indicating less tree-wide dispersion. The NTI is based on nearest phylogenetic taxon distance, and is more positive when there is more clustering at the branches than the null model, indicating less branch-tip dispersion.

## Results

**Figure 1:**
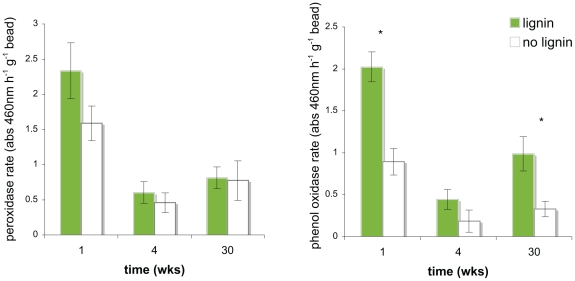
LIgnase activity of bio-traps after one, four, and thirty weeks in the field. These lignase assays are based on degradation of the lignin substrate analog L-dihydrophenylalanine (L-DOPA) with 0.3% hydrogen peroxide for peroxidase, and without for phenol oxidase. All assays were performed on fresh beads that had been in the ground 48 hours earlier. Enzyme activities reported as absorbance units per gram bead, and are means of six biological replicates with standard error bars shown, and with significance levels between treatments at each time point (p<0.05) are denoted by an asterisk (*).

Enzyme assays revealed significantly increased phenol oxidase activity in lignin-amended beads compared to unamended beads during the first and last collection period, and the same trend was seen for most sampling points for both phenol oxidase and peroxidase activities (Figure 1, Table S1). Over time there was significant decrease in peroxidase (p<0.0001) and near-significant decrease in phenol oxidase (p = 0.0675) enzyme activity (Table 1). At each time point, carbohydrate-active enzyme activities (beta-glucosidase, cellobiohydrolase, N-acetylglucosidase, and xylosidase) were always greater in the unamended controls compared to the amended beads (Table S1). Carbohydrate-active enzyme activities increased significantly over time and showed no overall difference between lignin-amended and unamended beads (Table 1).

**Table 1:** Summary of statistical analyses.

Factor		Lignin P-value	Lignin Trend	Time P-value	Time Trend
Enzyme activity	Phenol oxidase	<0.001	lignin>none	0.0675	T1>T2, T4
	Peroxidase	n.s.	n.a.	<0.0001	T1>>T2, T4
	Beta-glucosidase	n.s.	n.a.	<0.0001	T1, T2<T4
	Cellobiohydrolase	n.s.	n.a.	<0.0001	T1, T2<T4
	N-acetyl glucosaminidase	n.s.	n.a.	<0.0001	T1, T2<T4
	Xylosidase	n.s.	n.a.	<0.0001	T1, T2<T4
Q-PCR of total bacteria		n.s.	n.a.	<0.05	T1≤T2, T3≤T4
PhyloChip Microbial Community	Richness of bacteria	n.s.	n.a.	<0.001	T1, T4<T2, T3
	Net Relatedness Index (NRI)	n.s.	n.a.	0.0950	n.a.
	Nearest Taxon Index (NTI)	0.10	none>lignin	<0.01	T1>T2>T3, T4
Pyrosequencing Microbial Community	Richness of taxa	n.s.	n.a.	<0.0001	T1, T2<T3, T4
	Shannon's Diversity (H)	<0.05	lignin>none	<0.001	T1, T2<T3, T4

n.s. = not significant; n.a. = not applicable; NRI and NTI are measures of phylogenetic dispersion; see methods section for more detail.

Soils experienced fluctuating redox conditions throughout the 30 week study (Fig. S2A). Individual chambers ranged from a mean of 7.4 to 18.6% O_2_ and exhibited up to 18% cumulative probability of having less than 3% oxygen (Figure S2B), which is known to support anaerobic microbial metabolisms [16], [39]. The presence of elevated nitrous oxide and methane, both anaerobically mediated trace gases, was further evidence of abundant anaerobic microsites in the soil (Figure S2C).

Quantitative PCR (Q-PCR) of total bacterial cells demonstrated significantly more bacteria associated with the lignin-amended beads compared to the unamended beads in the beginning and end of the experiment (Table 2). There was also a significant increase in the bacterial colonization of the beads over time detectable by Q-PCR (Table 1).

**Table 2:** Q-PCR of total number of bacteria from bio-traps per gram bead.

T	time (weeks)	Lignin-amended	Unamended	p-value	Trend
T1	1 week	4.86E+03 (1.37E+03)	1.54E+03 (4.65E+02)	<0.05	Lignin>none
T2	4 weeks	1.51E+03 (5.31E+02)	4.39E+02 (8.99E+01)	<0.05	Lignin>none
T3	13 weeks	4.03E+03 (2.84E+03)	4.07E+03 (1.77E+03)	n.s.	n.a.
T4	30 weeks	1.75E+04 (1.38E+04)	8.10E+05 (7.93E+05)	n.s.	n.a.

n.s. = not significant; n.a. = not applicable; values are mean total bacteria (standard error, n = 4).

**Figure 2:**
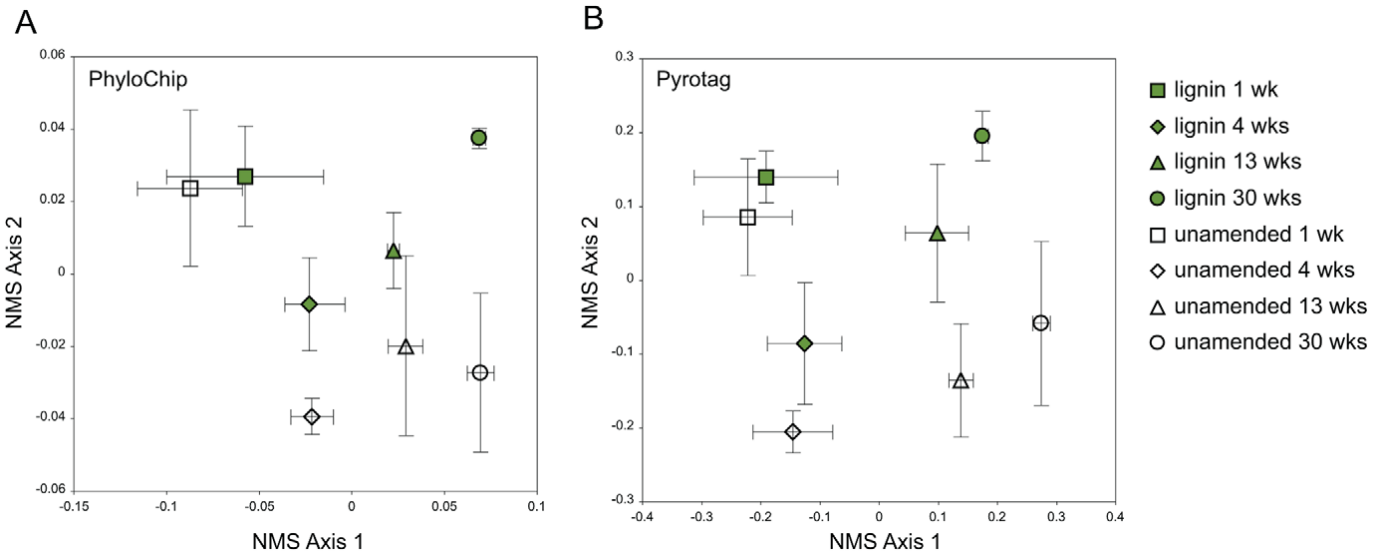
Microbial community analysis of bio-traps. Ordination is shown for (A) PhyloChip and (B) SSU rRNA pyrosequencing of microbial communities detected in lignin-amended and unamended biosep beads over time. For PhyloChip analysis there were 537 distinct bacterial taxa detected; for pyrosequencing there were 4,684 bacterial, archaeal, and eukaryotic taxa detected. In both analyses, ordination performed was nonmetric multidimensional scaling using Bray-Curtis distance measure, and mean ordination scores plus or minus standard error are shown based on four randomly chosen of the six biological replicates.

Microbial communities showed a significant separation of the lignin-amended bead community from the unamended bead community analyzed using amplicon pyrosequencing (Figure 2B; MRPP A = 0.0165, p<0.05) and PhyloChip (Figure 2A; MRPP A = 0.0135, p<0.10). The microbial community profile changed significantly over time in PhyloChip (MRPP A = 0.1952, p<0.001) and pyrosequencing analyses (MRPP A = 0.08899, p<0.001). Examining the SSU rRNA pyrosequencing data for each time point, there was a significant log linear relationship between the richness of taxa in the lignin-amended versus unamended beads (Figure S3); the slopes suggest that most groups were less abundant in lignin-amended compared to the unamended beads, and the magnitude of this difference increased over time.

The PhyloChip is designed based on a well-supported phylogenetic tree of Archaea and Bacteria [31], so a standardized measure of total phylogenetic distance was employed to estimate the effect of lignin and time on the microbial community phylogeny [38]. There was no significant change in the net relatedness index (NRI) over time or by amendment, but the nearest taxon index (NTI) was decreased significantly over time (p<0.01) and decreased in lignin-amended beads compared to unamended beads (p<0.10) (Table 1, Figure S4A). This suggests that with lignin amendment and over time, there was increased phylogenetic dispersion in the microbial communities. A plot of PhyloChip relatedness (NRI and NTI) by richness showed a distinct negative linear relationship, where increased richness was significantly correlated to increased branch-tip dispersion (Figure S4B). SSU rRNA amplicon pyrosequencing has the advantage of measuring normalized, absolute abundance of microbial taxa resulting in both richness and evenness estimates. We calculated Shannon's Diversity for the microbial communities as determined by pyrosequencing, which revealed a significant increase in diversity with lignin amendment and over time (Table 1, Figure S5). PhyloChip and pyrosequencing communities showed significantly increased richness with time, but not with lignin amendment (Table 1, Figure S6).

**Figure 3:**
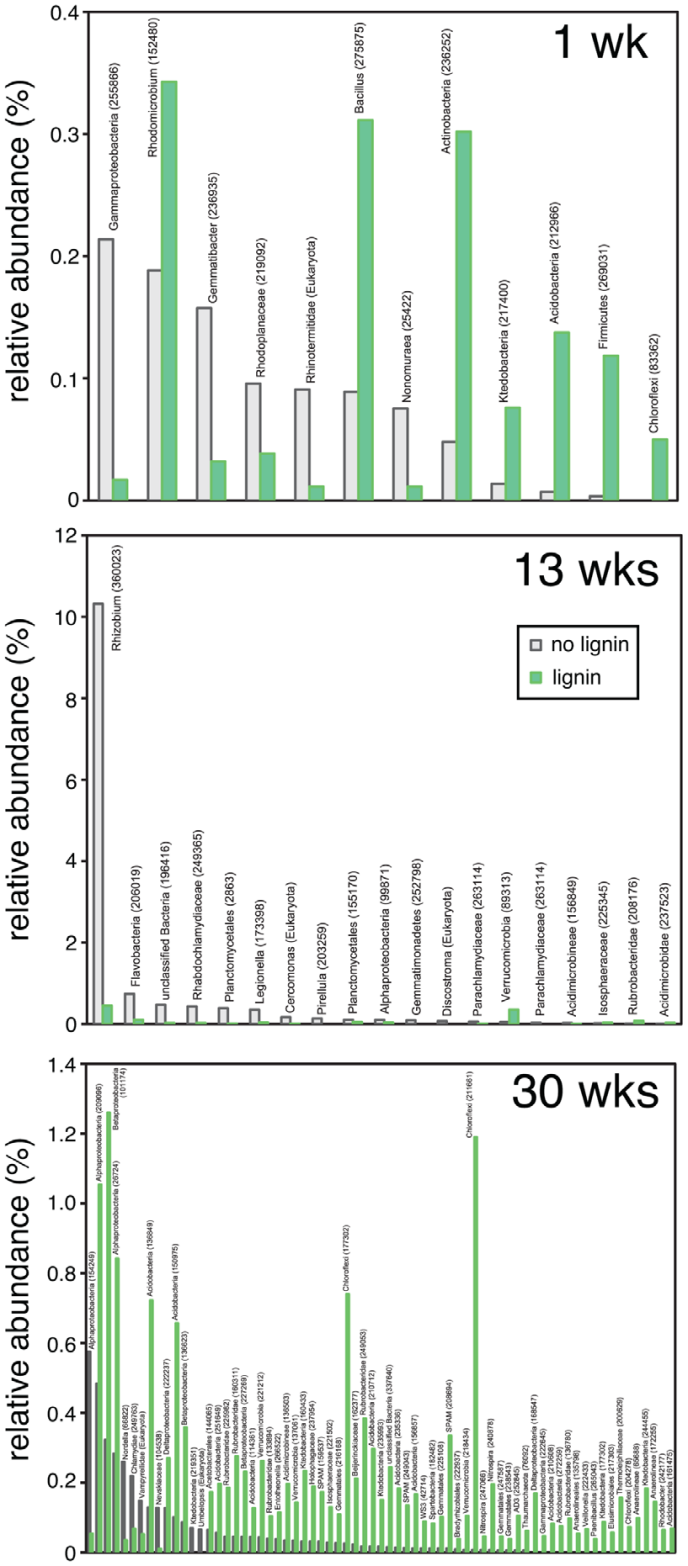
Rank-abundance comparison of SSU rRNA pyrosequencing results. A two-tailed t-test was performed to identify OTUs different between lignin-ameneded and unamended control beads. Several low-abundant members of the communities turned out to be significantly different. At T2 (4 weeks) there were no OTUs significantly different between beads. Taxa names are listed with greengenes taxon ID numbers in parentheses.

A summary of the taxa that are significantly different in the lignin-amended beads compared to unamended beads, broken down by time point, show differences in populations with lignin amendment and a community succession over time (Figure 3). Examining the PhyloChip taxa that had higher relative abundance at any time in the lignin-amended compared to unamended beads revealed dominance in the Acidobactera, Actinobacteria, Proteobacteria, and Verrucomicrobia (Table S2, S3). At the phylum level the dominant taxa found in the SSU rRNA pyrosequencing data agrees with PhyloChip data, with the additional finding that Planctomycetes and Eukaryota (not including fungi) were designated to be important members of the late stage bead communities, with Eukaryota significantly enriched in unamended compared to lignin amended beads (Tables S4, S5). There was no significant difference in fungal relative abundance between lignin-amended and unamended beads at any time point.

To identify taxa enriched in the lignin-amended beads we did a more detailed analysis on the microbial communities identified at the first time point after one week of incubation (T1), chosen because these samples had significantly higher phenol oxidase activities compared to the control beads; significantly lower cellulase enzyme activities; significantly lower richness and significantly higher numbers of bacteria as determined by Q-PCR. Based on PhyloChip results we performed a one-tailed, paired t-test for each identified taxon, and found a total of 39 taxa were significantly different between the lignin-amended beads and the controls, of which 38 were enriched on the lignin-amended beads (Table 3, Table S6). These taxa were affiliated with the phyla Acidobacteriales, Chloroflexi, Proteobacteria, Firmicutes, Verrucomicrobia including singletons belonging to other phyla. SSU rRNA amplicon pyrosequencing results showed that taxa closely related to Rhodomicrobium, Bacillus, Actinobacteria, Acidobacteria and Firmicutes are strongly enriched in the lignin beads after one week of incubation (Figure 3).

**Table 3:** Taxa significantly enriched in the T1 lignin-amended beads compared to unamended beads by PhyloChip analysis.

Phylum	Class	Total Taxa	Notes or Nearest Neighbor Taxa
Acidobacteria	Acidobacteriales	8	all in family Acidobacteraceae
	Unclassified	2	unclassified
Actinobacteria	Rubrobacterales	1	uranium mining waste clone
	Actinomycetales	1	*Arthrobacter ureafaciens*
Bacteroidetes	Sphingobacterales	1	uncultured environmental clone
BRC1	Unclassified	1	n.a.
Chloroflexi	Anaerolineae	3	uncultured environmental clone
	Dehalococcoidetes	1	uncultured environmental clone
	Unclassified	1	uncultured environmental clone
DSS1	Unclassified	1	dechlorinating consortium clone
Firmicutes	Clostridia	5	*Desulfosporosinus orientis*
Lentisphaerae	Unclassified	1	
Proteobacteria	a>Caulobacterales	2	*Caulobacter intermedius, Brevundimonas diminuta*
	d>Desulfovibrionales	1	*Desulfovibrio cuneatus*
	d>Syntrophobacterales	1	*Geobacter metallireducens*
	g>Enterobacteriales	1	uncultured environmental clone
SPAM	n.a.	1	*Leptospirillum ferrooxidans*
Spirochaetes	Spirochaetes	1	*Spironema culicis*
Verrucomicrobia	Verrucomicrobiae	4	*Prosthecobacter dejongeii*, uncultured environmental clones

n.a. = not applicable.

## Discussion

This study demonstrates that the lignin-amended biosep beads are an effective method for trapping soil populations with the specific capability of decomposing lignin. Substantial phenol oxidase and peroxidase accompanied by depressed carbohydrate-active enzyme activity and low microbial community richness after one week suggests the capture of a fairly specialized group of microorganisms adapted to the lignin-amended bead environment. There were a number of taxa that were dominant early on in the experiment and more abundant in lignin-amended than unamended bead communities, which presumably play a role in lignin decomposition in the soil. Bacteria known to break down lignin are concentrated in the Alphaproteobacteria, Gammaproteobacteria, and Actinomycetes [7]. Taxa in the class Alphaproteobacteria were the most dominant taxa from the earliest sampling time point, and significantly enriched in lignin beads compared to unamended beads. The Alphaproteobacteria picked up by the PhyloChip were closely related to *Caulobacter intermedius* and *Brevundimonas diminuta*, and these taxa are known catalase producers. *Caulobacter crescentis* is an obligate aerobe that produces catalase likely as protection from oxidative stress in late-stationary phase in culture [40]. *Rhodomicrobium* is an Alphaproteobacteria in the family Rhizobiales that was detected by the pyrosequencing analysis, and also a known purple non-sulfur bacterium. While taxa in this genus are able to link iron reduction and denitrification to photosynthesis [41], [42], their role in below-ground lignin decomposition likely involves their ability to fix nitrogen [19]. The Gammaproteobacteria we detected were in the Enterobacteraceae, closely related to the *Escherichia* spp. observed as lignin-degrading from the guts of wood-boring beetles [43]. Likewise the Actinomyces we observed were only distantly related to the well-characterized *Streptomyces viridosporus* and *Rhodococcus* spp. demonstrated to have lignin degrading activity [8], [44]. This departure is likely due to the many differences between tropical forest soils and the wood-eating insect gut environment where bacterial lignin degradation is well-documented. The lignin beads are going to pick up lignin-degrading bacteria as well as bacteria able to live on little to no carbon and also tolerate the presence of lignin and potentially toxic lignin byproducts of decomposition. However, the scarce availability of oxygen in these soils [16] accompanied by high amounts of iron and iron cycling [45] suggests potential non-oxidative mechanisms of lignin decomposition.

Frequent episodes of soil anoxia have been observed in these soils and are known to affect the microbial community [22], [46]. This fluctuating redox may facilitate the development of lignin-amended bead microbial communities with a diversity of mechanisms for decomposition. Fermentation is likely to play a role in anaerobic metabolism of complex carbon, evidenced by dominance of Bacilli in the phylum Firmicutes in the lignin-bead populations. Fermenters like *Enterobacteriaceae* has been observed to out-compete obligate anaerobes under similar conditions [47]. The Bacteroidetes bacterium *Flavobacterium meningosepticum* was isolated from soil and shown to not demonstrate catalase activity, though it has the ability to grow on phenolic, model lignin compounds as sole C and energy source [10]. Because labile carbon is limiting to soil microbes, we might expect lignin decomposition and assimilation to also be linked to denitrification, sulfate reduction, iron reduction, and methanogenesis, encompassing the range of metabolisms previously observed in these soils [21], [22].

Fungi are generally considered the main microbial decomposers of plant material [18], [19], [48], though we hypothesize that their role in tropical forest soils is diminished because of frequent anaerobic soil conditions [16]. Fungi were detected in the pyrotag data, but comprised a relatively small portion of the richness (<5.5%) and evenness (<5.8%), with more fungi in the unamended compared to the lignin-amended beads (Table S4, S5); phylogenetic information from metagenomic analysis supports this hypothesis [20]. We detected an abundance of acid-tolerant strains such as from the phylum Acidobacteria, which were enriched in the lignin-amended beads relative to the controls and have been found with decomposing fungi where perhaps their acid tolerance confers a competitive environment in the decomposing litter [49]. In anaerobic systems Actinobacteria and filamentous bacteria may play the role of fungi, producing phenol oxidases and peroxidases [19]. Members of the genus *Kocuria* (Actinobacteria) and *Staphylococcus* (Firmicutes) were previously described as phenol-degraders in soil [9] and were also detected in our beads.

There was a strong effect of time on the microbial community structure and function (Table 1, Figure 2) suggestive of microbial community succession. On a natural plant substrate, the initial community would have grown utilizing the more accessible cellulose and hemicellulose components before leaving the more resilient lignin compounds for the later stage communities [50]. In this respect, the beads are selecting directly for the organisms that are able to access the complex plant biomass that is characteristic of late stage decomposition. There was no cellulose substrate on the beads when they were buried, but these enzyme activities represent potential activities likely due to colonization of microbes. The increase in bacterial richness as well as cellulase activity towards the end of the experiment suggests that the lignin created relatively unfavorable conditions for the majority of the soil microbial community.

While the pyrosequencing and PhyloChip microarray microbial community profiles agreed well with each other, there were some differences at the species-level identification of lignin bead-associated microbial taxa due to the fact that these two methods assay microbial communities in very different ways. Though both begin with PCR amplification, the primers used are tailored to each method; for pyrosequencing, the universal primers are designed to capture as much of the bacteria, archaea, and eukaryota in any environmental sample [32], while the PhyloChip was designed around primers that capture as much of the 16S rRNA gene (bacteria and archaea only) as possible [51]. Though PCR amplification will distort relative abundances in mixed communities, pyrosequencing has the potential to more faithfully maintain relative abundances, while PhyloChip is sensitive enough to amplify and detect even quite rare members of the microbial community [52], [53]. Both methods are intended to provide a microbial community profile of specific environments, where the association with lignin beads suggests tolerance or utilization of lignin, though further studies are required to understand which taxa are responsible.

The lignin-baited biosep beads appear to be efficient bio-traps for capturing lignin-degrading microbial populations, baited with commercial alkali lignin and tested with L-DOPA, phenolic model lignin compounds which bear structural similarities to carbon compounds found in the environment like humics, lignin breakdown products and contaminants. The aromatic compounds benzoate, phenylpropionate and phenylacetate are produced as natural by-products in the anaerobic rhizosphere of rice field soil [54]. Some of these same compounds are formed in anaerobic fermentation reactions and can inhibit cell growth, biofuels production, or both [55]. So although we cannot directly assay the microbes active in late-stage decomposition through this method, we are able to identify and measure the activity of phenol-oxidase producing populations.

The data taken together suggest that the lignin had an adverse effect on all but a specific subset of the microbial community, and this select population is likely able to enzymatically access and assimilate carbon derived from the lignin. Phylogenetic analysis also demonstrated a significant increase in the diversity and clustering of the community on lignin-amended beads compared to unamended beads, suggesting that the lignin either directly created an chemical environment unfavorable to all but a small population of bacteria, or the taxa initially able to utilize the lignin had a competitive advantage and out competed later immigrant populations. The molecular mechanisms of this largely anaerobic lignin-degrading population are of interest and under investigation.

## Supporting Information

Figure S1 **Photographs of bio-traps.** These images show (A) Bio-Sep beads and (B) bio-traps made of slotted PVC to hold the beads.
(TIF)

Figure S2 **Gas concentrations in field chambers.** Oxygen concentration (A) and nitrous oxide, carbon dioxide, and methane (B) measured in the oxygen chambers in the field during the course of the experiment. The asterisks (*) show the times when samples were taken.
(TIF)

Figure S3 **Comparison of taxa detected by amplicon pyrosequencing in lignin-amended compared to unamended beads.** The correlations are shown with the R-square values, equations for linear fit as well as significance values. They are all significantly correlated, which would indicate no differences between lignin and no-lignin bead communities, however, from the R-square values it seems that there are some differences.
(TIF)

Figure S4 **Phylogenetic relatedness of PhyloChip microbial communities.** (A) Report of community relatedness by net relatedness index (NRI) or nearest taxon index (NTI). (B) Community relatedness is plotted as a function of community richness for PhyloChip microbial community analysis. The community analysis program phylocom was used to generate estimates of phylogenetic clustering in microbial communities using the net relatedness index (NRI), which is a measure of tree-wide phylogenetic dispersion, and nearest taxon index (NTI), which is a measure of branch-tip phylogenetic dispersion.
(TIF)

Figure S5 **Shannon's diversity index for microbial communitiesby SSU rRNA pyrosequencing.** The data are displayed as a box-and-whiskers plot.
(TIF)

Figure S6 **Richness of PhyloChip and pyrosequencing microbial communities.** Box plots display richness detected in lignin-amended and unamended biosep beads over time. For PhyloChip and pyrotag community richness, there was no significant trend between lignin-amended and unamended beads, though there was a significant effect of time.
(TIF)

Table S1 **Enzyme activity rates measured on fresh beads.**
(PDF)

Table S2 **PhyloChip richness of taxa from the lignin-amended and unamended beads.**
(PDF)

Table S3 **PhyloChip richness of taxa with significantly higher relative abundance in lignin-amended beads compared to unamended beads.**
(PDF)

Table S4 **Phylogenetic classification at the phylum level of bacterial taxa identified by SSU rRNA amplicon pyrosequencing.**
(PDF)

Table S5 **Phylogenetic classification of identified SSU rRNA sequence tags (pyrosequencing) based on phylum level.**
(PDF)

Table S6 **Taxonomy and nearest neighbor of 38 taxa significanlty enriched on lignin beads by PhyloChip.**
(PDF)
