# Supplementary figures and images for: Characterization of Trapped Lignin-Degrading Microbes in Tropical Forest Soil
Source: PLoS One. 2011 Apr 29;6(4):e19306. doi: 10.1371/journal.pone.0019306 (PMC3084812; doi:10.1371/journal.pone.0019306)

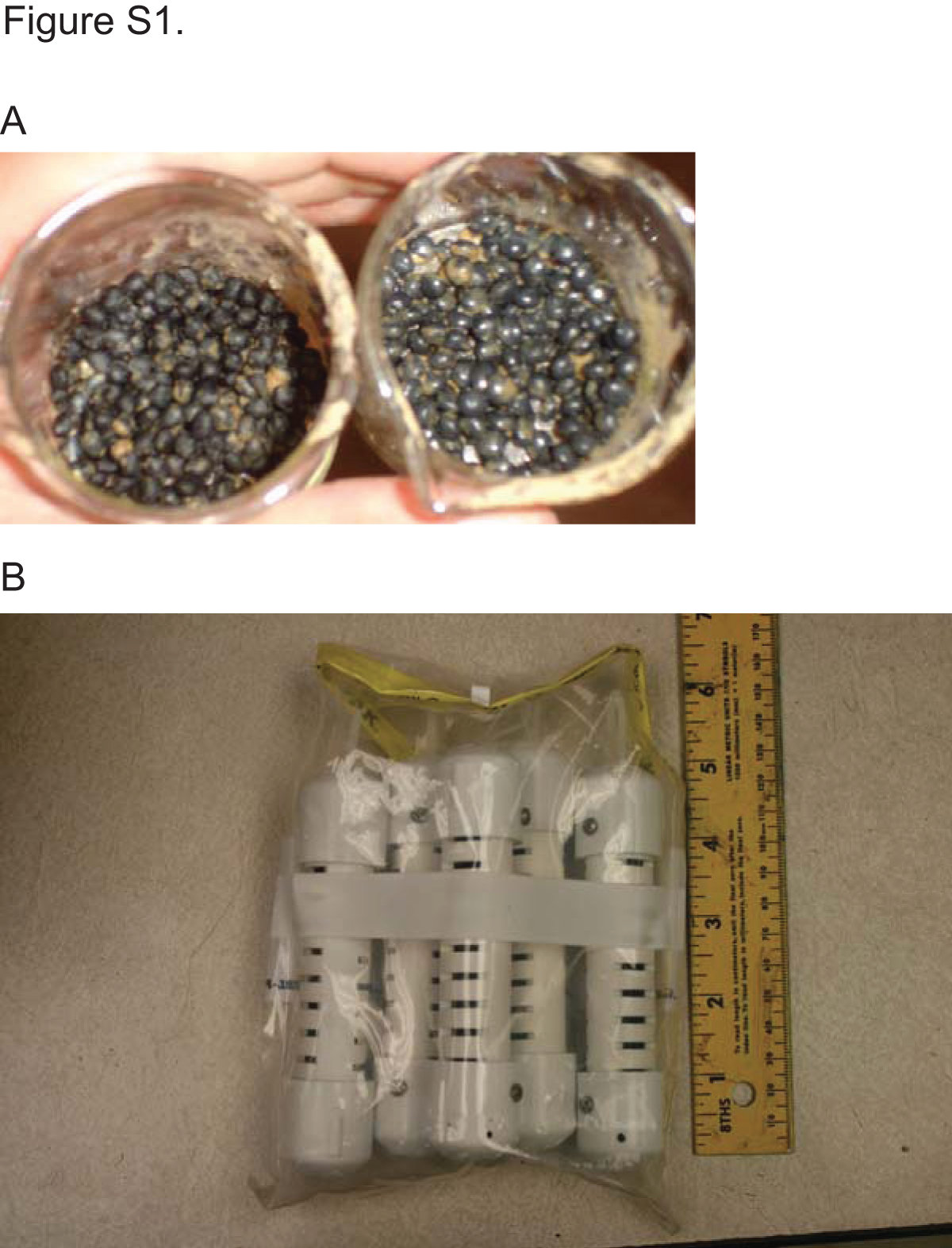

Supplement: Figure S1 — Photographs of bio-traps. These images show (A) Bio-Sep beads and (B) bio-traps made of slotted PVC to hold the beads. (TIF) [file pone.0019306.s001.tif]

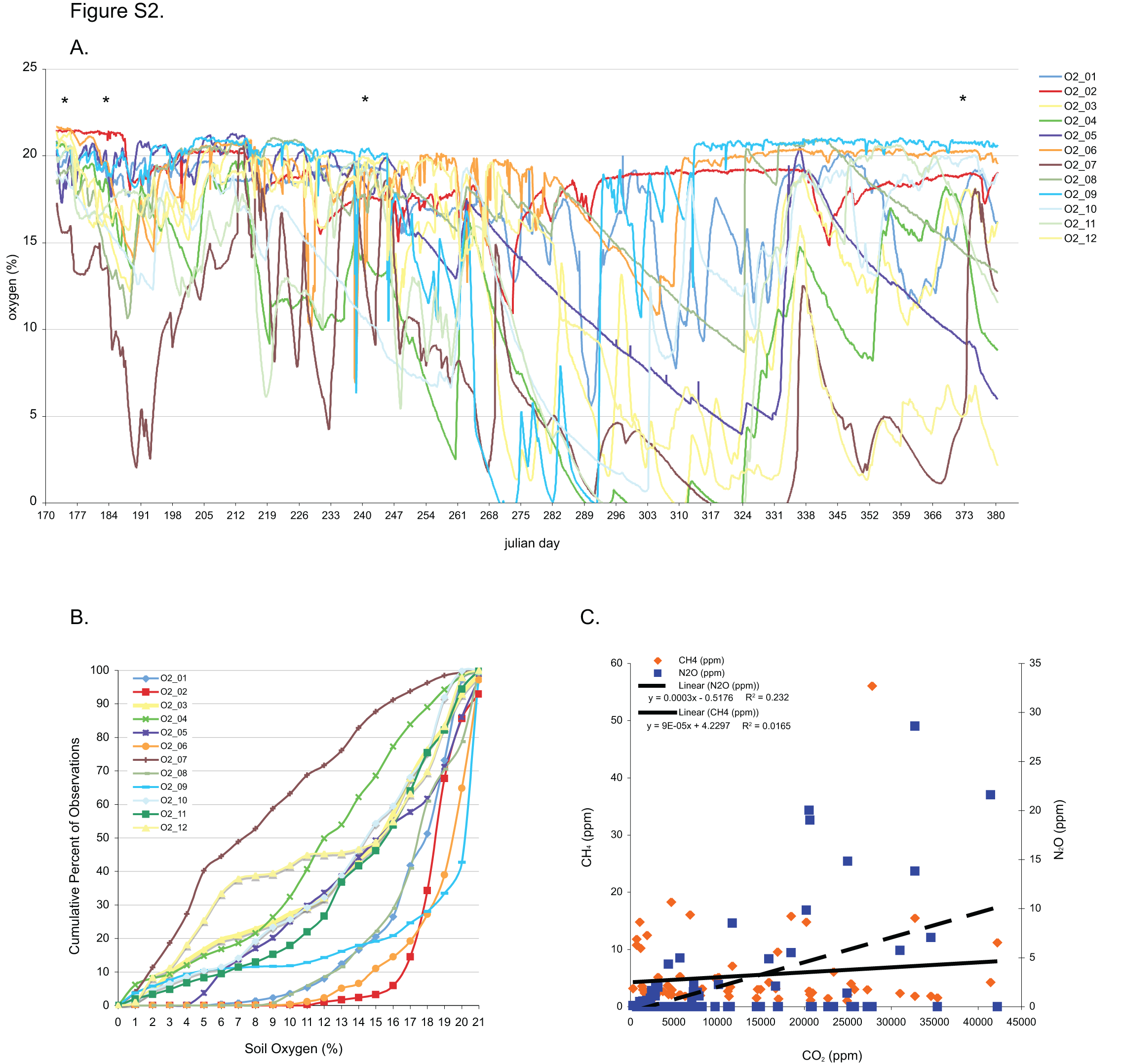

Supplement: Figure S2 — Gas concentrations in field chambers. Oxygen concentration (A) and nitrous oxide, carbon dioxide, and methane (B) measured in the oxygen chambers in the field during the course of the experiment. The asterisks (*) show the times when samples were taken. (TIF) [file pone.0019306.s002.tif]

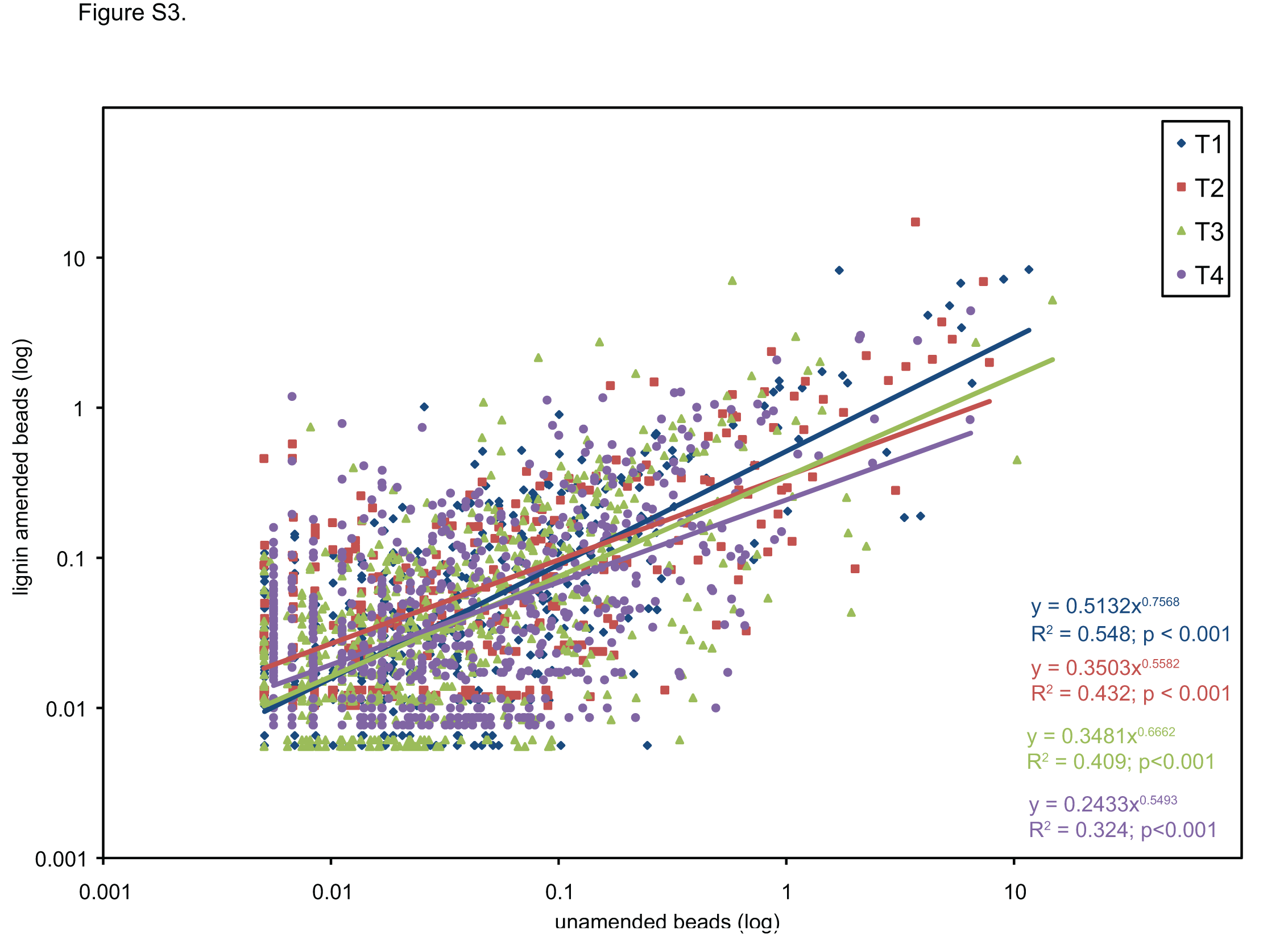

Supplement: Figure S3 — Comparison of taxa detected by amplicon pyrosequencing in lignin-amended compared to unamended beads. The correlations are shown with the R-square values, equations for linear fit as well as significance values. They are all significantly correlated, which would indicate no differences between lignin and no-lignin bead communities, however, from the R-square values it seems that there are some differences. (TIF) [file pone.0019306.s003.tif]

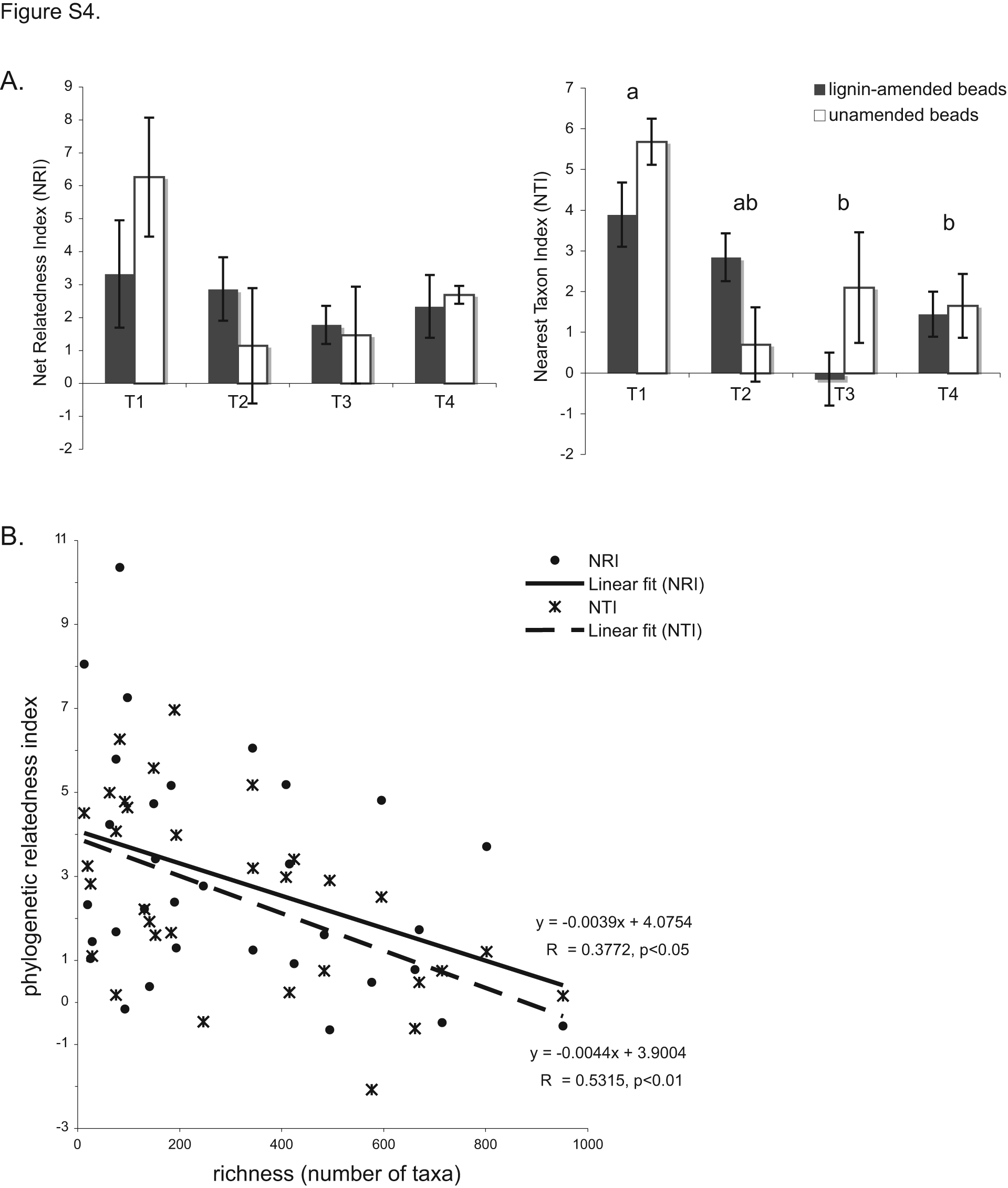

Supplement: Figure S4 — Phylogenetic relatedness of PhyloChip microbial communities. (A) Report of community relatedness by net relatedness index (NRI) or nearest taxon index (NTI). (B) Community relatedness is plotted as a function of community richness for PhyloChip microbial community analysis. The community analysis program phylocom was used to generate estimates of phylogenetic clustering in microbial communities using the net relatedness index (NRI), which is a measure of tree-wide phylogenetic dispersion, and nearest taxon index (NTI), which is a measure of branch-tip phylogenetic dispersion. (TIF) [file pone.0019306.s004.tif]

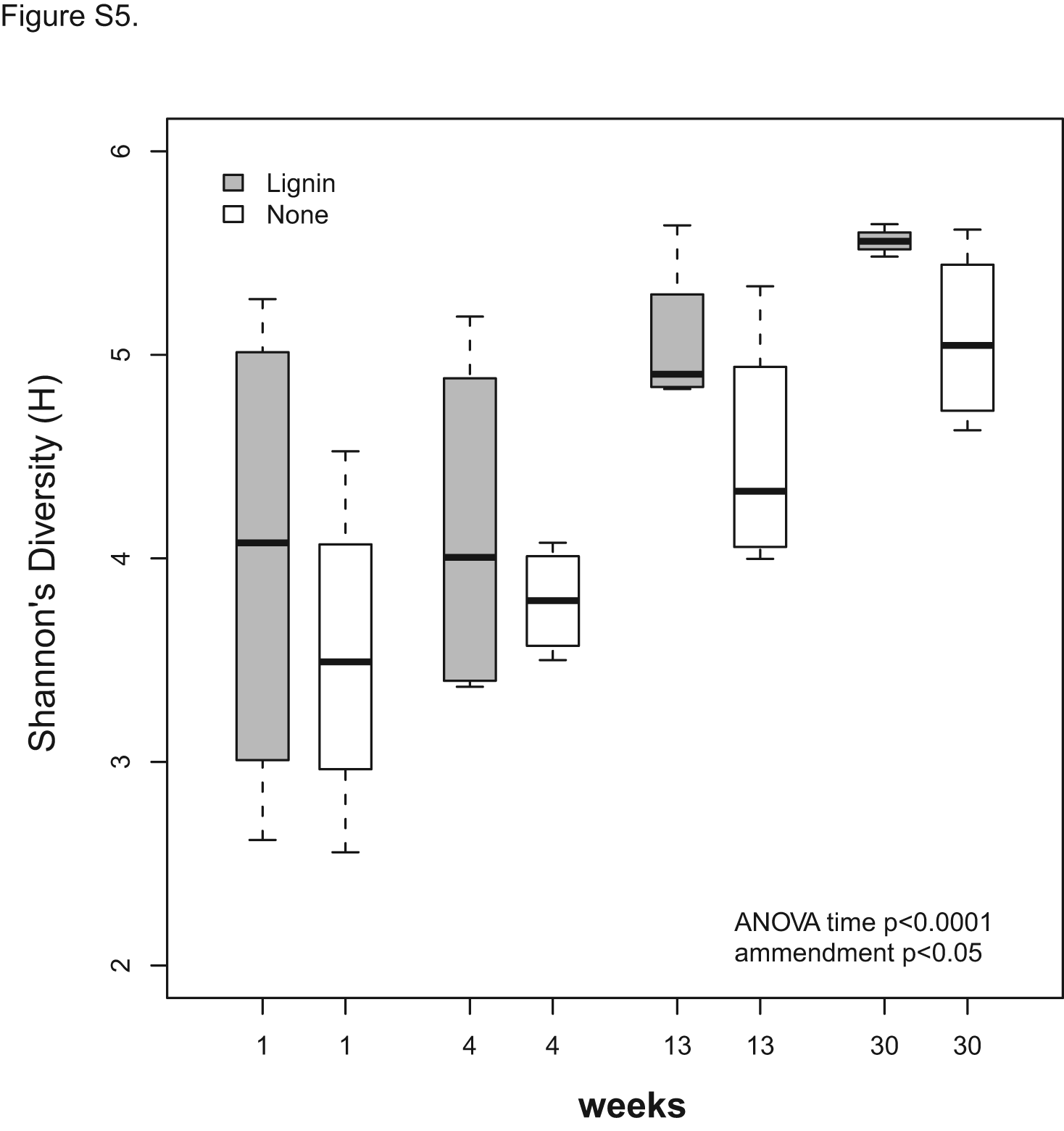

Supplement: Figure S5 — Shannon's diversity index for microbial communitiesby SSU rRNA pyrosequencing. The data are displayed as a box-and-whiskers plot. (TIF) [file pone.0019306.s005.tif]

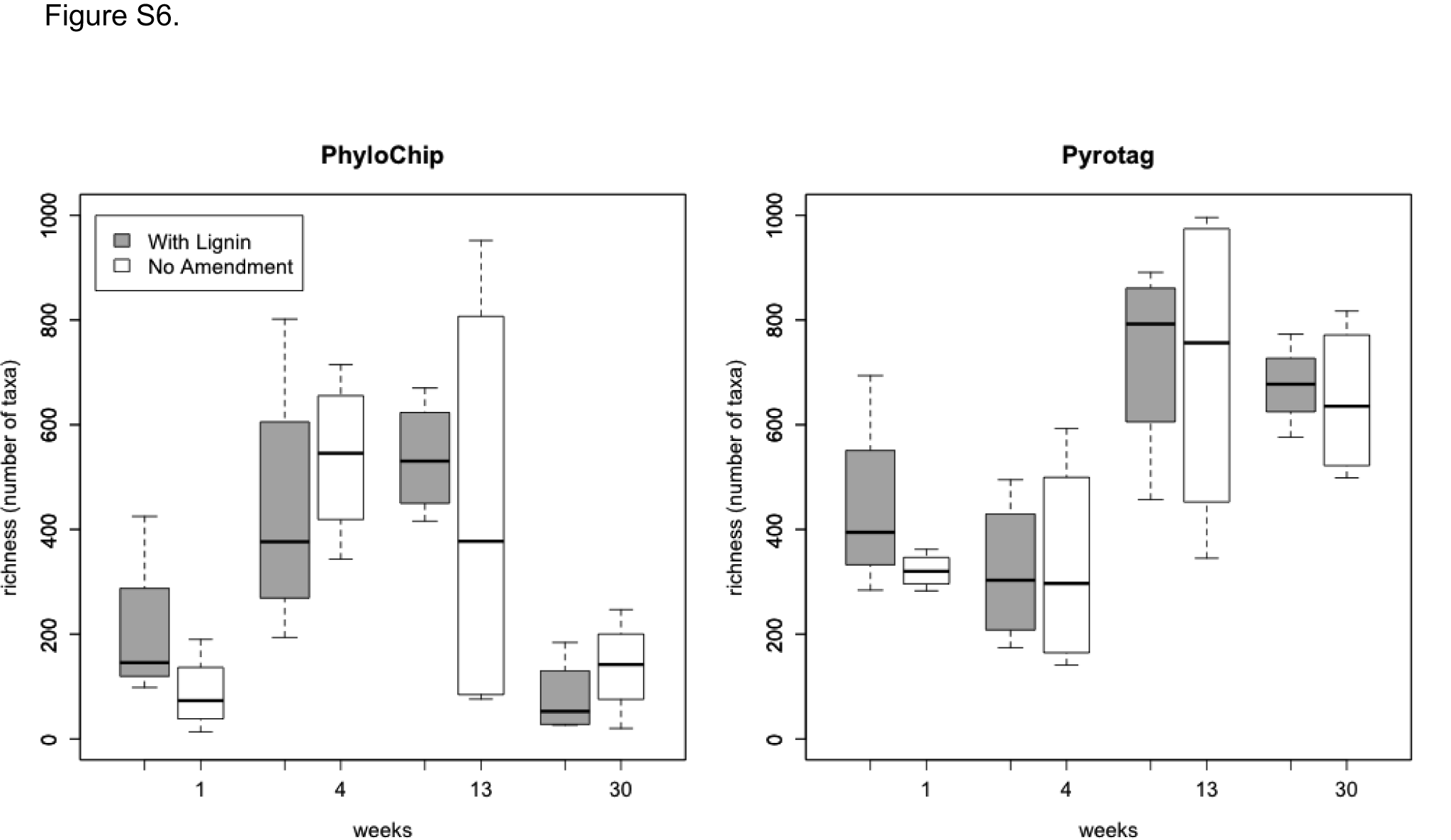

Supplement: Figure S6 — Richness of PhyloChip and pyrosequencing microbial communities. Box plots display richness detected in lignin-amended and unamended biosep beads over time. For PhyloChip and pyrotag community richness, there was no significant trend between lignin-amended and unamended beads, though there was a significant effect of time. (TIF) [file pone.0019306.s006.tif]
