# Supplementary material for: Characterization of Trapped Lignin-Degrading Microbes in Tropical Forest Soil
Source: PLoS One. 2011 Apr 29;6(4):e19306. doi: 10.1371/journal.pone.0019306 (PMC3084812; doi:10.1371/journal.pone.0019306)
Supplement: Table S1 — Enzyme activity rates measured on fresh beads. (PDF) [file pone.0019306.s007.pdf]

Table S1. Enzyme activities measured on fresh beads.

	Lignin-amended beads	Unamended beads	p-value
Phenol oxidase (mmol L-DOPA h^{-1} g^{-1})			
T1 (1 week)	1.449 +/- 0.227	1.189 +/- 0.125	n.s.
T2 (4 weeks)	0.044 +/- 0.012	0.018 +/- 0.013	n.s.
T4 (30 weeks)	0.375 +/- 0.08	0.118 +/- 0.033	<0.05
Peroxidase (mmol L-DOPA h^{-1} g^{-1})			
T1 (1 week)	0.139 +/- 0.021	0.085 +/- 0.012	<0.05
T2 (4 weeks)	0.021 +/- 0.004	0.007 +/- 0.001	<0.05
T4 (30 weeks)	D	ND	
b-Glucosidase (mmol MUB h^{-1} g^{-1})			
T1 (1 week)	96.11 +/- 7.9	116.11 +/- 2.06	0.05
T2 (4 weeks)	138.77 +/- 5.57	145.65 +/- 13.95	n.s.
T4 (30 weeks)	316.37 +/- 28.78	381.8 +/- 56.51	0.05
Cellobiohydrolase (mmol MUB h^{-1} g^{-1})			
T1 (1 week)	100.39 +/- 7.08	116.46 +/- 2.06	n.s.
T2 (4 weeks)	152.84 +/- 9.66	155.05 +/- 15.15	n.s.
T4 (30 weeks)	339.35 +/- 24.13	400.51 +/- 49.97	n.s.
N-acetyl glucosaminidase (mmol MUB h^{-1} g^{-1})			
T1 (1 week)	96.82 +/- 7.75	116.75 +/- 2.07	<0.05
T2 (4 weeks)	110.41 +/- 16.9	110.38 +/- 19.59	n.s.
T4 (30 weeks)	317.51 +/- 30.51	347.05 +/- 68.11	<0.05
Xylanase (mmol MUB h^{-1} g^{-1})			
T1 (1 week)	97.17 +/- 7.63	116.29 +/- 2.06	<0.05
T2 (4 weeks)	151.38 +/- 9.52	152.61 +/- 15.34	n.s.
T4 (30 weeks)	331.61 +/- 24.18	396.18 +/- 50.51	<0.05
