# Supplementary material for: Characterization of Trapped Lignin-Degrading Microbes in Tropical Forest Soil
Source: PLoS One. 2011 Apr 29;6(4):e19306. doi: 10.1371/journal.pone.0019306 (PMC3084812; doi:10.1371/journal.pone.0019306)
Supplement: Table S2 — PhyloChip richness of taxa from the lignin-amended and unamended beads. (PDF) [file pone.0019306.s008.pdf]

Table S2. PhyloChip richness of taxa from the lignin-amended and unamended bugtraps.

	T1		T2		T3		T4	
	lignin	no lignin	lignin	no lignin	lignin	no lignin	lignin	no lignin
Proteobacteria	131	71	291	365	322	248	46	92
Firmicutes	12	3	27	30	39	53	3	5
Acidobacteria	13	3	22	22	30	22	10	8
Bacteroidetes	11	3	18	31	22	22	5	7
Actinobacteria	11	1	19	17	30	34	1	1
Verrucomicrobia	8	3	10	10	11	8	5	6
Chloroflexi	4	1	8	12	15	13	1	4
Unclassified	4	2	9	11	13	9	2	4
Cyanobacteria	2	1	4	5	7	5	1	2
Spirochaetes	1	0	3	4	6	4	1	1
Synergistes	1	0	4	4	5	3	1	2
Planctomycetes	1	0	2	5	5	4	0	2
Gemmatimonadetes	1	0	3	4	5	4	0	1
OP10	1	0	2	2	3	1	0	0
SPAM	1	0	1	2	2	1	1	1
Nitrospira	1	0	1	1	2	1	1	1
marine group A	1	0	1	1	1	1	1	1
Chlorobi	0	0	1	2	2	2	0	0
Deinococcus-Thermus	0	0	1	2	2	1	0	0
Lentisphaerae	0	0	1	1	2	2	0	0
OP3	0	0	1	1	2	1	0	1
BRC1	1	0	1	1	2	1	0	0
Natronoanaerobium	1	0	1	1	2	2	0	0
NC10	0	0	1	1	2	1	0	0
TM7	0	0	1	2	2	1	0	0
OP9/JS1	0	0	1	1	1	1	0	0
WS3	0	0	1	1	1	1	0	0
Thermodesulfobacteria	0	0	1	1	1	1	0	0
Caldithrix	0	0	0	1	1	1	0	0
AD3	0	0	0	1	1	1	0	0
LD1PA	0	0	1	1	1	0	0	0
DSS1	0	0	1	0	1	0	0	0
TM6	0	0	1	0	0	1	0	0
Chlamydiae	0	0	0	1	1	0	0	0
OP8	0	0	0	0	0	1	0	0
Aquificae	0	0	0	0	0	0	0	0
