# Supplementary material for: Characterization of Trapped Lignin-Degrading Microbes in Tropical Forest Soil
Source: PLoS One. 2011 Apr 29;6(4):e19306. doi: 10.1371/journal.pone.0019306 (PMC3084812; doi:10.1371/journal.pone.0019306)
Supplement: Table S3 — PhyloChip richness of taxa with significantly higher relative abundance in lignin-amended beads compared to unamended beads. (PDF) [file pone.0019306.s009.pdf]

Table S3. PhyloChip richness of taxa with significantly higher relative abundance in lignin-amended bug traps compared to unamended bug traps.

	T1		T2		T3		T4	
	lignin	no lignin	lignin	no lignin	lignin	no lignin	lignin	no lignin
Proteobacteria	27	1	61	73	121	68	24	16
Acidobacteria	34	10	57	53	75	52	24	19
Actinobacteria	4	0	24	21	40	50	1	1
Verrucomicrobia	12	5	17	15	14	12	10	10
Unclassified	7	2	17	15	23	13	4	6
Firmicutes	4	0	12	12	21	27	1	0
Chloroflexi	2	0	10	11	18	17	0	1
Synergistes	2	0	7	6	8	4	2	4
SPAM	3	0	5	6	7	4	2	3
Gemmatimonadetes	2	0	6	3	6	4	1	2
OP10	1	0	4	5	6	3	1	1
Lentisphaerae	1	0	3	4	6	4	0	0
BRC1	1	0	2	3	4	2	1	0
NC10	1	0	2	2	4	2	0	0
Bacteroidetes	0	0	1	2	3	2	0	0
Natronoanaerobium	0	0	2	0	2	2	0	0
OP9/JS1	0	0	1	2	1	2	0	0
Caldithrix	0	0	0	1	2	1	0	0
Chlorobi	0	0	0	1	0	1	0	0
