# Supplementary material for: Characterization of Trapped Lignin-Degrading Microbes in Tropical Forest Soil
Source: PLoS One. 2011 Apr 29;6(4):e19306. doi: 10.1371/journal.pone.0019306 (PMC3084812; doi:10.1371/journal.pone.0019306)
Supplement: Table S4 — Phylogenetic classification at the phylum level of bacterial taxa identified by SSU rRNA amplicon pyrosequencing. (PDF) [file pone.0019306.s010.pdf]

Table S4. Phylogenetic classification at the phylum level of bacterial taxa identified by SSU rRNA amplicon pyrosequencing. Values indicate **"richness"** (number of unique taxa detected for each phylum) given as percentage. Phyla contributing ≥7.5% are in **bold**, and ≥ are also **underlined**. Average values are from n=4 beads. n.d. = not detected.

	T1		T2		T3		T4	
	no lignin	lignin	no lignin	lignin	no lignin	lignin	no lignin	lignin
Archaea	0.2 ±0.3	0.2 ±0.4	0.1 ±0.2	0.1 ±0.3	0.2 ±0.3	0.1 ±0.1	0.3 ±0.1	0.6 ±0.3
ABY1 OD1	n.d.	n.d.	n.d.	n.d.	0.1 ±0.1	0.1 ±0.2	0.1 ±0.1	0.2 ±0.3
Acidobacteria	8.6 ±2.1	7.6 ±1.3	4.7 ±2.0	5.0 ±3.7	5.6 ±1.6	7.5 ±2.5	5.6 ±1.7	8.5 ±0.9
Actinobacteria	11.7 ±2.5	12.9 ±1.7	7.9 ±2.3	8.0 ±1.3	8.1 ±0.9	8.9 ±2.2	6.7 ±1.3	9.4 ±1.4
AD3	0.1 ±0.2	0.5 ±0.2	0.1 ±0.1	0.1 ±0.1	0.1 ±0.1	0.2 ±0.2	0.1 ±0.2	0.3 ±0.2
Bacteroidetes	6.1 ±4.5	3.9 ±1.5	8.0 ±4.1	6.4 ±2.0	4.7 ±0.6	6.3 ±0.7	4.7 ±1.1	4.4 ±0.3
BRC1	n.d.	n.d.	0.2 ±0.4	n.d.	n.d.	0.2 ±0.1	0.2 ±0.2	0.1 ±0.3
Caldithrix	n.d.	n.d.	n.d.	n.d.	n.d.	0.1 ±0.1	n.d.	0.0 ±0.1
Chlamydiae	1.2 ±0.7	1.7 ±0.9	0.9 ±1.0	3.1 ±1.9	3.7 ±1.1	2.1 ±1.1	5.0 ±2.4	2.9 ±1.1
Chlorobi	0.0	0.1 ±0.1	0.4 ±0.7	0.3 ±0.3	0.4 ±0.3	0.6 ±0.3	0.2 ±0.0	0.5 ±0.1
Chloroflexi	9.5 ±1.2	14.4 ±1.5	3.9 ±3.5	4.9 ±1.8	5.6 ±1.7	7.7 ±1.9	4.7 ±3.0	9.7 ±1.4
CV51	n.d.	n.d.	n.d.	n.d.	n.d.	n.d.	0.1 ±0.1	0.1 ±0.1
Cyanobacteria	0.4 ±0.3	0.3 ±0.2	0.0 ±0.1	0.5 ±0.5	0.7 ±0.4	0.5 ±0.4	0.9 ±0.2	0.3 ±0.3
Desulfitobacter	n.d.	n.d.	n.d.	n.d.	n.d.	n.d.	0.0 ±0.1	n.d.
Elev_16S_998	n.d.	n.d.	n.d.	n.d.	n.d.	n.d.	n.d.	0.1 ±0.1
Elusimicrobia	0.2 ±0.3	0.2 ±0.2	0.1 ±0.1	0.3 ±0.5	0.2 ±0.4	0.5 ±0.5	0.4 ±0.3	0.8 ±0.3
Entotheonella	0.2 ±0.2	0.2 ±0.2	0.1 ±0.3	0.1 ±0.1	0.1 ±0.1	0.1 ±0.1	0.1 ±0.1	0.1 ±0.0
FCPS706	0.2 ±0.2	0.2 ±0.1	0.1 ±0.1	0.2 ±0.2	0.1 ±0.1	0.1 ±0.1	0.1 ±0.1	0.1 ±0.0
Fibrobacteres	n.d.	n.d.	n.d.	n.d.	n.d.	0.1 ±0.1	n.d.	n.d.
Firmicutes	6.4 ±1.6	8.5 ±1.1	3.7 ±2.4	3.6 ±1.5	3.5 ±1.1	3.5 ±1.0	3.3 ±1.0	4.0 ±4.0
GAL15	0.3 ±0.3	0.4 ±0.2	0.1 ±0.2	0.1 ±0.1	0.2 ±0.1	0.1 ±0.0	0.2 ±0.2	0.1 ±0.1
Gemmatimonadetes	0.9 ±0.5	0.7 ±0.2	0.8 ±0.6	1.0 ±0.9	1.9 ±0.4	1.7 ±0.3	1.6 ±0.7	1.6 ±0.4
GN02	n.d.	n.d.	n.d.	0.1 ±0.1	n.d.	0.1 ±0.1	0.0 ±0.1	0.0 ±0.1
Lentisphaerae	n.d.	n.d.	n.d.	n.d.	n.d.	n.d.	0.0 ±0.1	n.d.
Marine group A	n.d.	n.d.	n.d.	n.d.	n.d.	0.0 ±0.1	n.d.	n.d.

Table S4, cont'd	T1		T2		T3		T4	
	no lignin	lignin	no lignin	lignin	no lignin	lignin	no lignin	lignin
MBMPE71	n.d.	n.d.	n.d.	n.d.	n.d.	0.0 ±0.1	n.d.	n.d.
NC10	0.1 ±0.2	0.3 ±0.1	n.d.	0.1 ±0.1	0.1 ±0.1	0.1 ±0.1	0.1 ±0.2	0.2 ±0.1
Nitrospirae	0.1 ±0.2	0.4 ±0.6	0.2 ±0.3	0.3 ±0.5	0.4 ±0.3	0.9 ±0.7	0.3 ±0.3	1.2 ±0.4
NKB19	n.d.	n.d.	n.d.	n.d.	n.d.	0.1 ±0.1	0.0 ±0.1	0.0 ±0.1
O1aA90	n.d.	n.d.	n.d.	n.d.	n.d.	n.d.	n.d.	0.1 ±0.1
O339	0.1 ±0.2	n.d.	n.d.	n.d.	n.d.	n.d.	0.0 ±0.1	n.d.
OP3	n.d.	n.d.	n.d.	n.d.	0.0 ±0.1	n.d.	0.0 ±0.1	0.0 ±0.1
OP5	n.d.	n.d.	n.d.	n.d.	n.d.	0.0 ±0.1	0.0 ±0.1	0.1 ±0.1
OP10	0.5 ±1.1	0.4 ±0.1	0.1 ±0.1	0.7 ±0.7	0.2 ±0.2	0.9 ±0.7	0.2 ±0.2	0.7 ±0.2
OP11	n.d.	0.1 ±0.1	n.d.	0.2 ±0.3	0.3 ±0.4	0.6 ±0.4	0.3 ±0.4	1.6 ±0.7
Planctomycetes	5.4 ±1.0	5.3 ±2.2	4.2 ±3.3	5.4 ±0.9	9.9 ±1.0	8.6 ±0.2	8.3 ±1.6	9.5 ±1.7
<u>Alphaproteobacteria</u>	<u>16.2 ±2.9</u>	<u>14.2 ±0.7</u>	<u>25.0 ±8.1</u>	<u>20.5 ±3.3</u>	<u>14.9 ±4.0</u>	<u>13.7 ±3.0</u>	<u>13.3 ±0.7</u>	<u>11.1 ±0.9</u>
Betaproteobacteria	7.1 ±2.1	6.2 ±1.9	9.2 ±1.6	7.2 ±1.9	4.3 ±2.2	5.4 ±1.8	4.4 ±0.5	4.2 ±0.5
Gammaproteobacteria	6.0 ±1.4	4.9 ±1.4	8.6 ±2.9	6.8 ±2.8	8.0 ±1.7	5.2 ±1.0	9.2 ±3.7	6.1 ±1.2
Deltaproteobacteria	4.1 ±1.0	3.8 ±1.3	4.9 ±2.0	4.5 ±1.2	5.5 ±1.6	7.8 ±0.9	5.2 ±1.9	6.9 ±1.0
Epsilonproteobacteria	n.d.	n.d.	n.d.	n.d.	n.d.	0.0 ±0.1	n.d.	0.0 ±0.1
SC3	n.d.	0.1 ±0.2	n.d.	0.2 ±0.3	0.1 ±0.1	0.1 ±0.1	0.1 ±0.1	0.3 ±0.2
SC4	n.d.	n.d.	n.d.	n.d.	0.0 ±0.1	0.1 ±0.1	n.d.	0.0 ±0.1
SHA-95	n.d.	n.d.	n.d.	n.d.	n.d.	n.d.	0.0 ±0.1	0.0 ±0.1
SM2F11	n.d.	n.d.	n.d.	0.2 ±0.2	0.0 ±0.1	0.1 ±0.2	0.1 ±0.2	0.2 ±0.1
SPAM	0.5 ±0.3	0.8 ±0.2	0.3 ±0.2	0.4 ±0.5	0.4 ±0.1	0.6 ±0.3	0.2 ±0.1	0.7 ±0.1
Spirochaetes	0.2 ±0.2	0.0 ±0.1	0.4 ±0.3	0.5 ±0.5	0.1 ±0.1	0.3 ±0.1	0.4 ±0.3	0.2 ±0.2
SR1	n.d.	n.d.	n.d.	0.1 ±0.1	n.d.	n.d.	n.d.	n.d.
Synergistetes	n.d.	n.d.	n.d.	n.d.	n.d.	n.d.	0.0 ±0.1	n.d.
Thermi	0.4 ±0.2	0.2 ±0.2	0.1 ±0.1	n.d.	0.1 ±0.1	0.1 ±0.1	n.d.	n.d.
Thermotogae	n.d.	n.d.	n.d.	n.d.	0.0 ±0.1	n.d.	n.d.	n.d.
TM6	0.9 ±0.6	0.6 ±0.3	0.6 ±0.7	1.0 ±1.0	2.7 ±0.6	1.1 ±0.6	2.9 ±2.0	1.5 ±0.3
TM7	0.1 ±0.3	0.2 ±0.4	0.3 ±0.3	1.2 ±0.6	0.6 ±0.2	0.4 ±0.3	0.4 ±0.3	0.3 ±0.3
Verrucomicrobia	2.3 ±0.6	1.9 ±0.2	2.5 ±1.0	2.7 ±1.5	2.0 ±0.4	3.2 ±1.3	2.2 ±0.7	3.3 ±0.4
VHS-B5-50	n.d.	0.1 ±0.1	n.d.	n.d.	0.1 ±0.1	n.d.	n.d.	n.d.

Table S4, cont'd	T1		T2		T3		T4	
	no lignin	lignin	no lignin	lignin	no lignin	lignin	no lignin	lignin
WCHB1-27	n.d.	n.d.	n.d.	0.1 ±0.1	n.d.	n.d.	n.d.	0.1 ±0.1
WPS-2	0.4 ±0.1	0.4 ±0.1	0.1 ±0.3	0.1 ±0.3	0.1 ±0.1	0.2 ±0.2	0.1 ±0.1	0.3 ±0.2
WS3	n.d.	0.2 ±0.2	n.d.	0.2 ±0.2	0.1 ±0.1	0.3 ±0.3	0.0 ±0.1	0.2 ±0.1
WS5	n.d.	n.d.	n.d.	n.d.	n.d.	0.0 ±0.1	n.d.	0.0 ±0.1
WS6	n.d.	n.d.	n.d.	n.d.	n.d.	0.1 ±0.1	n.d.	n.d.
ZB2	n.d.	n.d.	n.d.	0.2 ±0.3	0.3 ±0.3	0.2 ±0.2	0.7 ±0.6	0.4 ±0.6
ZB3	n.d.	n.d.	n.d.	n.d.	n.d.	n.d.	0.0 ±0.1	n.d.
Fungi	3.0 ±1.2	2.2 ±0.8	2.7 ±1.7	3.9 ±2.0	4.0 ±0.8	2.1 ±0.7	5.5 ±2.0	2.0 ±0.8
Eukaryota	4.3 ±1.9	5.1 ±2.7	7.0 ±4.8	8.2 ±1.5	9.3 ±2.1	6.3 ±4.3	10.2 ±2.8	4.2 ±1.1
Chloroplasts	0.1 ±0.1	n.d.	0.0 ±0.1	n.d.	0.1 ±0.1	0.1 ±0.1	0.0 ±0.1	0.0 ±0.1
Unclassified	2.1 ±0.5	0.9 ±0.4	2.6 ±1.1	2.0 ±0.9	1.0 ±0.4	0.6 ±0.4	1.1 ±0.3	0.5 ±0.1
