# Supplementary material for: Characterization of Trapped Lignin-Degrading Microbes in Tropical Forest Soil
Source: PLoS One. 2011 Apr 29;6(4):e19306. doi: 10.1371/journal.pone.0019306 (PMC3084812; doi:10.1371/journal.pone.0019306)
Supplement: Table S5 — Phylogenetic classification of identified SSU rRNA sequence tags (pyrosequencing) based on phylum level. (PDF) [file pone.0019306.s011.pdf]

Table S5. Phylogenetic classification of identified SSU rRNA sequence tags (pyrosequencing) based on phylum level. Values indicate **“evenness”** (number of sequence tags identified for each phylum) given as percentage. Phyla contributing ≥10% are in bold. Average values are from n=4 beads. n.d. = not detected.

	T1		T2		T3		T4	
	no lignin	lignin	no lignin	lignin	no lignin	lignin	no lignin	lignin
Archaea	n.d.	0.1 ±0.1	n.d.	n.d.	0.0 ±0.1	0.0 ±0.1	0.1 ±0.0	0.5 ±0.3
ABY1 OD1	n.d.	n.d.	n.d.	n.d.	n.d.	n.d.	0.1 ±0.1	0.1 ±0.1
Acidobacteria	2.6 ±1.7	4.3 ±3.0	0.8 ±0.4	2.5 ±2.8	3.1 ±3.0	6.2 ±3.7	3.5 ±2.8	10.6 ±1.1
Actinobacteria	5.7 ±4.5	10.6 ±6.5	3.3 ±1.6	6.0 ±1.9	5.5 ±2.1	7.8 ±3.2	6.1 ±3.4	10.4 ±2.5
AD3	n.d.	0.1 ±0.1	n.d.	n.d.	n.d.	0.1 ±0.0	n.d.	0.1 ±0.1
Bacteroidetes	2.3 ±2.3	2.2 ±1.8	5.3 ±4.9	3.6 ±2.1	4.0 ±0.8	4.4 ±2.7	4.3 ±2.1	2.6 ±0.6
BRC1	n.d.	n.d.	0.0 ±0.1	n.d.	n.d.	0.1 ±0.1	0.0 ±0.1	0.0 ±0.1
Caldithrix	n.d.	n.d.	n.d.	n.d.	n.d.	0.0 ±0.1	n.d.	n.d.
Chlamydiae	0.1 ±0.0	0.3 ±0.2	0.1 ±0.2	1.0 ±0.8	4.5 ±1.8	0.8 ±0.5	7.2 ±6.9	1.7 ±1.2
Chlorobi	n.d.	n.d.	1.5 ±1.8	0.1 ±0.1	0.1 ±0.1	0.6 ±0.8	0.1 ±0.1	0.2 ±0.1
Chloroflexi	1.3 ±0.5	6.4 ±4.8	0.4 ±0.3	1.7 ±1.7	2.3 ±2.6	5.0 ±3.0	2.5 ±2.3	8.8 ±2.8
Cyanobacteria	n.d.	n.d.	n.d.	0.1 ±0.1	0.4 ±0.2	0.2 ±0.2	0.5 ±0.3	0.1 ±0.1
Elusimicrobia	n.d.	n.d.	n.d.	0.2 ±0.3	0.0 ±0.1	0.2 ±0.3	0.1 ±0.1	0.3 ±0.2
Entotheonella	n.d.	0.1 ±0.1	0.0 ±0.1	0.1 ±0.1	n.d.	0.1 ±0.1	n.d.	0.1 ±0.0
FCPS706	n.d.	0.1 ±0.1	n.d.	n.d.	n.d.	n.d.	n.d.	0.1 ±0.0
Firmicutes	7.0 ±7.8	4.2 ±3.4	1.2 ±1.3	1.7 ±1.6	1.0 ±0.6	1.7 ±0.3	1.4 ±0.8	1.8 ±0.3
GAL15	0.1 ±0.1	0.3 ±0.3	n.d.	0.1 ±0.1	0.1 ±0.1	0.1 ±0.1	0.1 ±0.1	0.1 ±0.1
Gemmatimonadetes	0.2 ±0.1	0.1 ±0.1	0.2 ±0.1	0.3 ±0.3	0.9 ±0.3	0.8 ±0.3	1.3 ±0.8	0.7 ±0.3
GN02	n.d.	n.d.	n.d.	n.d.	n.d.	0.0 ±0.1	n.d.	n.d.
NC10	n.d.	0.1 ±0.1	n.d.	n.d.	0.0 ±0.1	0.2 ±0.1	n.d.	0.5 ±0.4
Nitrospirae	n.d.	0.2 ±0.3	0.0 ±0.1	0.2 ±0.2	0.1 ±0.1	0.3 ±0.2	0.1 ±0.1	0.7 ±0.2
OP10	0.1 ±0.1	0.1 ±0.0	n.d.	0.2 ±0.2	0.0 ±0.1	0.4 ±0.2	0.1 ±0.1	0.2 ±0.1
OP11	n.d.	n.d.	n.d.	n.d.	0.0 ±0.1	0.3 ±0.3	0.1 ±0.1	0.6 ±0.3
Planctomycetes	0.8 ±0.3	1.4 ±1.1	1.2 ±1.0	1.2 ±0.6	4.0 ±0.7	2.8 ±0.6	4.7 ±0.4	4.8 ±0.8
Alphaproteobacteria	13.2 ±7.5	12.4 ±8.3	29.8 ±11.1	22.9 ±11.0	40.2 ±16.8	26.0 ±9.5	34.5 ±4.0	26.1 ±3.9
Betaproteobacteria	38.4 ±6.2	36.6 ±19.4	28.1 ±6.7	36.9 ±13.9	13.7 ±8.5	21.0 ±7.6	7.5 ±3.2	10.7 ±1.9

Table S5, cont'd	T1		T2		T3		T4	
	no lignin	lignin	no lignin	lignin	no lignin	lignin	no lignin	lignin
<u>Gammaproteobacteria</u>	<u>18.3 ±18.6</u>	<u>14.2 ±11.4</u>	8.4 ±6.6	5.5 ±3.0	4.6 ±1.1	3.2 ±1.6	6.4 ±2.1	4.1 ±1.4
Deltaproteobacteria	0.7 ±0.6	1.1 ±0.8	1.7 ±1.7	2.6 ±1.6	2.0 ±1.1	5.8 ±3.2	2.4 ±1.7	5.1 ±2.1
SC3	n.d.	n.d.	n.d.	0.1 ±0.1	n.d.	n.d.	n.d.	0.1 ±0.1
SM2F11	n.d.	n.d.	n.d.	n.d.	n.d.	0.1 ±0.1	0.0 ±0.1	0.1 ±0.0
SPAM	0.1 ±0.1	0.2 ±0.1	n.d.	0.1 ±0.2	0.2 ±0.2	0.4 ±0.3	0.1 ±0.0	0.8 ±0.2
Spirochaetes	n.d.	n.d.	0.1 ±0.1	0.1 ±0.1	0.1 ±0.1	0.1 ±0.0	0.1 ±0.0	0.1 ±0.1
SR1	n.d.	n.d.	n.d.	0.0 ±0.1	n.d.	n.d.	n.d.	n.d.
Thermi	0.2 ±0.3	0.1 ±0.1	n.d.	n.d.	n.d.	n.d.	n.d.	n.d.
TM6	0.1 ±0.1	0.1 ±0.1	1.0 ±2.0	0.3 ±0.2	1.4 ±1.1	0.4 ±0.3	2.0 ±1.9	0.6 ±0.3
TM7	n.d.	0.1 ±0.1	0.1 ±0.1	0.3 ±0.1	0.1 ±0.0	0.1 ±0.1	0.1 ±0.1	0.1 ±0.1
Verrucomicrobia	0.6 ±0.4	0.6 ±0.4	0.6 ±0.3	1.0 ±1.0	0.8 ±0.6	1.7 ±0.8	1.7 ±0.6	2.7 ±0.2
WCHB1-27	n.d.	n.d.	n.d.	n.d.	n.d.	n.d.	n.d.	0.1 ±0.0
WPS-2	0.1 ±0.1	0.1 ±0.0	n.d.	n.d.	n.d.	0.1 ±0.0	n.d.	0.1 ±0.1
WS3	n.d.	n.d.	n.d.	n.d.	0.0 ±0.1	0.1 ±0.1	n.d.	0.1 ±0.1
ZB2	n.d.	n.d.	n.d.	n.d.	0.0 ±0.1	0.2 ±0.1	0.4 ±0.4	0.2 ±0.3
Fungi	5.8 ±10.4	0.7 ±0.4	3.0 ±2.4	3.7 ±4.0	5.3 ±4.3	1.6 ±1.2	3.6 ±0.9	1.4 ±0.9
Alveolata	0.3 ±0.2	0.6 ±0.1	3.4 ±3.9	0.6 ±0.8	0.4 ±0.2	2.5 ±3.8	1.3 ±0.7	0.3 ±0.2
Cercozoa	0.3 ±0.1	0.5 ±0.7	2.8 ±2.8	3.0 ±2.9	1.3 ±0.4	2.5 ±2.9	2.2 ±0.8	0.8 ±0.8
Euglenozoa	0.1 ±0.1	1.2 ±2.0	0.2 ±0.2	0.5 ±0.9	0.2 ±0.1	0.1 ±0.1	0.7 ±1.2	0.1 ±0.2
Heterolobosea	n.d.	n.d.	0.7 ±1.3	0.3 ±0.3	0.3 ±0.2	0.0 ±0.1	0.3 ±0.3	0.1 ±0.1
Eukaryota (others)	0.6 ±0.6	0.4 ±0.4	1.0 ±0.7	1.9 ±0.9	1.8 ±0.6	1.2 ±0.9	3.4 ±1.9	1.1 ±0.5
Chloroplasts	n.d.	n.d.	n.d.	n.d.	n.d.	n.d.	0.0 ±0.1	n.d.
Unclassified	0.7 ±0.4	0.4 ±0.4	4.6 ±3.2	1.0 ±0.5	1.1 ±0.5	0.6 ±0.8	0.6 ±0.3	0.4 ±0.1
