# Supplementary material for: Characterization of Trapped Lignin-Degrading Microbes in Tropical Forest Soil
Source: PLoS One. 2011 Apr 29;6(4):e19306. doi: 10.1371/journal.pone.0019306 (PMC3084812; doi:10.1371/journal.pone.0019306)
Supplement: Table S6 — Taxonomy and nearest neighbor of 38 taxa significanlty enriched on lignin beads by PhyloChip. (PDF) [file pone.0019306.s012.pdf]

Table S6. Taxonomy and nearest neighbor of 38 taxa significanlty enriched on lignin beads by PhyloChip.

Phylum	Class	Order	Family	OTU_ID	GenBank ID
Acidobacteria	Acidobacteria	Acidobacteriales	Acidobacteriaceae	6345	AJ534634.1
Acidobacteria	Acidobacteria	Acidobacteriales	Acidobacteriaceae	6356	AF523985.1
Acidobacteria	Acidobacteria	Acidobacteriales	Acidobacteriaceae	6359	AF529322.1
Acidobacteria	Acidobacteria	Acidobacteriales	Acidobacteriaceae	6366	AJ292578.1
Acidobacteria	Acidobacteria	Acidobacteriales	Acidobacteriaceae	6368	AF200698.1
Acidobacteria	Acidobacteria	Acidobacteriales	Acidobacteriaceae	6378	D26171.1
Acidobacteria	Acidobacteria	Acidobacteriales	Acidobacteriaceae	6412	AF047646.1
Acidobacteria	Acidobacteria	Acidobacteriales	Acidobacteriaceae	6423	AF523979.1
Acidobacteria	Acidobacteria-6	Unclassified	Unclassified	500	Z95717.1
Acidobacteria	Acidobacteria-7	Unclassified	Unclassified	588	AJ009461.1
Actinobacteria	Actinobacteria	Actinomycetales	Unclassified	1405	X80744.1
Actinobacteria	Actinobacteria	Rubrobacterales	Rubrobacteraceae	1843	AJ536866.1
Bacteroidetes	Sphingobacteria	Sphingobacteriales	Flexibacteraceae	6297	AF502211.1
BRC1	Unclassified	Unclassified	Unclassified	118	AY218548.1
Chloroflexi	Anaerolineae	Chloroflexi-1f	Unclassified	765	AJ278167.1
Chloroflexi	Anaerolineae	Unclassified	Unclassified	205	AJ532729.1
Chloroflexi	Anaerolineae	Unclassified	Unclassified	946	AY216458.1
Chloroflexi	Dehalococcoidetes	Unclassified	Unclassified	2339	AJ519643.1
Chloroflexi	Unclassified	Unclassified	Unclassified	2523	AJ347055.1
DSS1	Unclassified	Unclassified	Unclassified	38	AJ306783.1
Firmicutes	Clostridia	Clostridiales	Lachnospiraceae	2931	AB088994.1
Firmicutes	Clostridia	Clostridiales	Lachnospiraceae	4510	AB089034.1
Firmicutes	Clostridia	Clostridiales	Peptococc/Acidaminococc	242	AJ493052.1
Firmicutes	Clostridia	Clostridiales	Unclassified	2324	
Firmicutes	Clostridia	Clostridiales	Unclassified	3476	
Lentisphaerae	Unclassified	Unclassified	Unclassified	10330	AF507900.1
Proteobacteria	Alphaproteobacteria	Caulobacterales	Caulobacteraceae	6781	AB023784.1
Proteobacteria	Alphaproteobacteria	Caulobacterales	Caulobacteraceae	6968	AB021415.1
Proteobacteria	Deltaproteobacteria	Desulfovibrionales	Desulfovibrionaceae	10016	AB089110.1
Proteobacteria	Deltaproteobacteria	Syntrophobacterales	Syntrophobacteraceae	10021	AJ519630.1
Proteobacteria	Gammaproteobacteria	Enterobacteriales	Enterobacteriaceae	433	AF523903.1
SPAM	Unclassified	Unclassified	Unclassified	738	AJ532725.1
Spirochaetes	Spirochaetes	Spirochaetales	Spirochaetaceae	6558	AF166259.1
Unclassified	Unclassified	Unclassified	Unclassified	6355	
Verrucomicrobia	Unclassified	Unclassified	Unclassified	288	U60012.1
Verrucomicrobia	Verrucomicrobiae	Verrucomicrobiales	Verrucomicrobia subdivision 7	446	AY114322.1
Verrucomicrobia	Verrucomicrobiae	Verrucomicrobiales	Verrucomicrobia subdivision 5	530	AY114334.1
Verrucomicrobia	Verrucomicrobiae	Verrucomicrobiales	Verrucomicrobiaceae	1024	AY244959.1
